# Bioactive 30-Noroleanane Triterpenes from the Pericarps of *Akebia trifoliata*

**DOI:** 10.3390/molecules19044301

**Published:** 2014-04-04

**Authors:** Jing Wang, Qiao-Lin Xu, Meng-Fei Zheng, Hui Ren, Ting Lei, Ping Wu, Zhong-Yu Zhou, Xiao-Yi Wei, Jian-Wen Tan

**Affiliations:** 1 Key Laboratory of Plant Resources Conservation and Sustainable Utilization, South China Botanical Garden, Chinese Academy of Sciences, Guangzhou 510650, China; 2 Biotechnology Division, Guangdong Academy of Forestry, Guangzhou 510520, China; 3 College of Life Sciences, University of Chinese Academy of Sciences, Beijing 100049, China

**Keywords:** *Akebia trifoliata*, nortriterpenoids, bacteriostatic activity, cytotoxicity, *α*-glucosidase inhibitor

## Abstract

Two new 30-noroleanane triterpenes, 2*α*,3*β*,20*α*-trihydroxy-30-norolean-12-en-28-oic acid (**1**), 2*α*,3*β*-dihydroxy-23-oxo-30-norolean-12,20(29)-dien-28-oic acid (**2**), were isolated from the pericarps of *Akebia trifoliata*, together with four known ones, 3*β*-akebonoic acid (**3**), 2*α*,3*β*-dihydroxy-30-noroleana-12,20(29)-dien-28-oic acid (**4**), 3*α*-akebonoic acid (**5**) and quinatic acid (**6**). Their structures were established on the basis of detailed spectroscopic analysis, and they were all isolated from the pericarps of *A. trifoliata* for the first time. Compounds **3**−**6** showed *in vitro* bacteriostatic activity against four assayed Gram-positive bacterial strains. In particular **3** showed antibacterial activity toward *MRSA* with a MIC value 25 μg/mL, which was more potent than kanamycin (MIC 125 μg/mL). No compounds showed antibacterial activity toward the three Gram-negative bacteria tested. Compounds **4** and **5** showed interesting *in vitro* growth inhibitory activity against human tumor A549 and HeLa cell lines, with IC_50_ values ranging from 8.8 and 5.6 μM, respectively. Compounds **1**, **2**, **5** and **6** were further revealed to show significant *in vitro* *α*-glucosidase inhibitory activity with IC_50_ values from 0.035 to 0.367 mM, which were more potent than the reference compound acarbose (IC_50_ 0.409 mM).

## 1. Introduction

**Figure 1:**
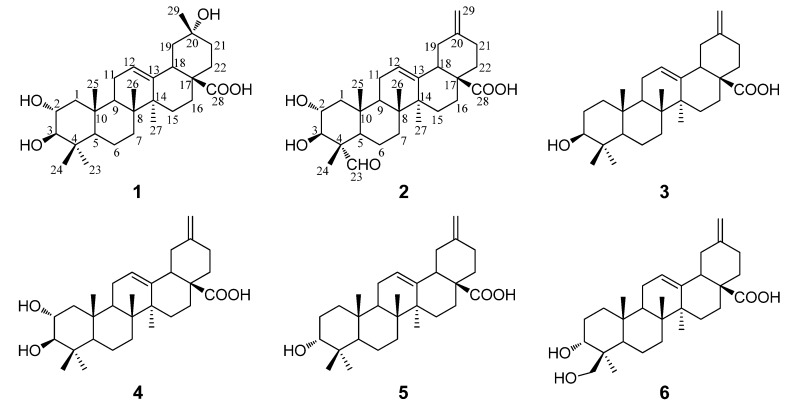
Chemical structures of compounds **1**−**6**.

*Akebia trifoliata* (Thunb.) Koidz., belonging to the family Lardizabalaceae, is a perennial, woody liana mainly distributed in the eastern part of Asia [1]. The fruit of *A. trifoliata*, commonly called ‘Bayuezha’ in China, has long been consumed by the local people as a delicious food [2]. The air-dried stems and fruits of *A. trifoliata* have also traditionally been used in China as an antiphlogistic, antineoplastic and diuretic agent for hundreds of years [3,4]. Previously, phytochemical studies have revealed many triterpenes and triterpene saponins from *A. trifoliata* [5,6,7], and phenolics and lignans were also reported from this species [8,9]. However, those studies were mainly concentrated on the stems and seldom focused on the pericarps, although it is highly possible that the pericarps of *A. trifoliata* would be a promising source of functional bioactive natural products [10,11,12]. Very recently, a phytochemical study revealed fifteen compounds, including eleven noroleanane triterpenoids from a methanol extract of pericarps of *A. trifoliata* [13], suggesting noroleanane triterpenoids to be characteristic in the pericarps of this species. Noroleanane triterpenoids are a group of novel natural products bearing skeletons with one or two carbons missing from the basic oleanane skeleton, which are so far only discovered in a small group of plants and some of them have been revealed to show significant bioactivities [14]. With the aim of clarifying the uncharacterized bioactive compounds in the pericarps of *A. trifoliata*, a phytochemical study on the pericarps of this species was carried out, whereby two new (compounds **1**–**2**) and four known 30-noroleanane triterpenes (compounds **3**–**6**) were obtained (Figure 1). Herein reported are the isolation and structure elucidation of these compounds, as well as their bacteriostatic, cytotoxic and *α*-glucosidase inhibitory activities.

## 2. Results and Discussion

**Figure 2:**
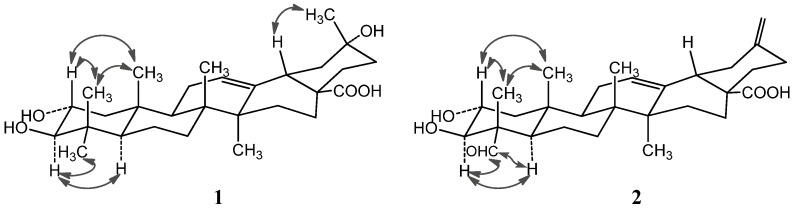
Important NOESY correlations of compounds **1** and **2**.

**Figure 3:**
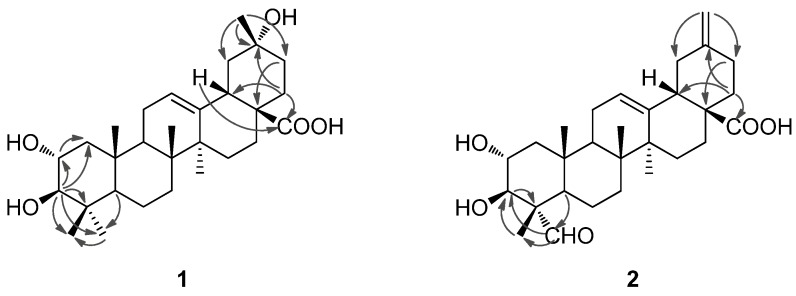
Selected HMBC correlations of compounds **1** and **2**.

Compound **1** was obtained as a white, amorphous powder. Positive mode HRESIMS indicated a molecular formula of C_29_H_46_O_5_ ([M+Na]^+^ *m*/*z* 497.3235, calcd. 497.3237), implying seven degrees of unsaturation. The ^1^H-NMR spectrum of **1** (Table 1) showed signals readily recognized for six tertiary methyl groups at *δ* 1.32 (3H, Me-29), 1.06 (3H, Me-27), 1.03 (3H, Me-23), 0.82 (3H, Me-25), 0.81 (3H, Me-24) and 0.76 (3H, Me-26). In addition, proton signals for two oxymethines at *δ* 3.77 (1H, H-2) and 3.09 (1H, H-3), and an olefinic proton at *δ* 5.26 (1H, H-12) were also observed. The ^13^C-NMR and DEPT spectra (Table 1) of **1** supported the above analysis, which showed 29 carbons including six methyls (*δ*_C_ 29.2, 17.6, 16.7, 17.4, 25.9 and 25.6), nine methylenes, six methines [including one olefinic methine at *δ* 122.5 (C-12), two oxy-methines at *δ* 68.5 (C-2) and 83.7 (C-3)], and eight quaternary carbons [including an olefinic quaternary carbon at *δ* 144.3 (C-13), a carboxyl carbon at *δ* 179.9 (C-28), and an oxy-quaternary carbon at *δ* 69.8 (C-20)]. These above findings accounted for two of the seven degrees of unsaturation, suggesting that **1** is a pentacyclic nortriterpenoid. Comparison of the NMR data of **1** with those of maslinic acid indicated that they were structurally closely related, with the major difference of a germinal methyl group attached at C-20 in maslinic acid being replaced by a hydroxyl group in **1** [15]. This assignment was consistent with the molecular formula of **1** and in accord with the significant change of the chemical shift value for C-20 from *δ*_C_ 30.8 in maslinic acid to *δ*_C_ 69.7 in **1**. The location of the hydroxyl group at C-20 was furhter supported by HMBC correlations from *δ*_H_ 1.32 (H_3_-29) to *δ*_C_ 47.6 (C-19), 69.8 (C-20), 36.1 (C-21) and from *δ*_H_ 1.75 (H_2_-22) to C-20. In the NOESY spectrum, the important NOE correlation between Me-29 (*δ*_H_ 1.32) and H-18 (*δ*_H _2.98) was observed, which supported the hydroxyl group at C-20 was *α*-orientation (Figure 2). In addition, the HMBC correlations (Figure 3) from *δ*_H_ 1.75 (H-22) and 2.98 (H-18) to *δ*_C_ 179.9 (C-28) supported the location of the carboxyl group at C-17. The HMBC correlations from *δ*_H_ 5.26 (H-12) to *δ*_C_ 42.1 (C-14), 44.3 (C-18) and 47.9 (C-9) supported the location of double bond at C-12(13). The HMBC correlations from *δ*_H_ 3.77 (H-2) to *δ*_C_ 47.7 (C-1) and *δ*_C_ 83.7 (C-3), and from *δ*_H_ 3.09 (H-3) to *δ*_C_ 47.7 (C-1), 68.5 (C-2), 39.7 (C-4), 29.2 (C-23) and 17.6 (C-24) supported that each of C-2 and C-3 was attached with a hydroxyl group. 

**Table 1:** ^1^H-NMR and ^13^C-NMR data for compounds **1** and **2**, *δ* in ppm and *J* in Hz.

No.	*δ*_C_ (1)	*δ*_H_ (1)	*δ*_C_ (2)	*δ*_H_ (2)
1	47.7 CH_2_	1.95 (dd, 11.8, 3.7), 0.97 (m)	47.8 CH_2_	2.29 (dd, 12.0, 4.2), 1.41 (m)
2	68.5 CH	3.77 (td, 11.8, 9.5, 3.7)	67.9 CH	4.24 (td, 12.0, 9.3, 4.2)
3	83.7 CH	3.09 (d, 9.5)	77.0 CH	4.06 (d, 9.3)
4	39.7 C	―	56.5 C	―
5	55.8 CH	0.77 (m)	47.9 CH	1.64 (dd, 10.5, 1.8)
6	18.7 CH_2_	1.41 (m), 1.23 (m)	20.5 CH_2_	1.50 (m), 1.10 (m)
7	33.1 CH_2_	1.34 (m), 1.13 (m)	32.3 CH_2_	1.51 (m), 1.21 (m)
8	39.7 C	―	39.7 C	―
9	47.9 CH	1.53 (m)	47.8 CH	1.89 (m)
10	38.4 C	―	38.2 C	―
11	23.8 CH_2_	2.03 (m), 1.75 (m)	23.6 CH_2_	2.07 (d, 11.1), 1.97 (dd, 11,1, 3.4)
12	122.5 CH	5.26 (t, 3.2)	122.5 CH	5.48 (t, 3.4)
13	144.3 C	―	144.1 C	―
14	42.1 C	―	42.0 C	―
15	28.2 CH_2_	1.86 (m), 0.98 (m)	28.1 CH_2_	2.17 (m), 1.99 (m)
16	23.7 CH_2_	2.03 (m), 1.76 (m)	23.7 CH_2_	2.18 (m), 1.93 (m)
17	46.6 C	―	46.9 C	―
18	44.3 CH	2.98 (dd, 14.0, 3.7)	47.4 CH	3.23 (dd, 13.5, 4.6)
19	47.6 CH_2_	2.10 (bt, 14.0), 1.57 (m)	41.8 CH_2_	2.64 (bt, 13.5), 2.25 (m)
20	69.8 C	―	148.9 C	―
21	36.1 CH_2_	1.73 (m), 1.57 (m)	30.2 CH_2_	2.30 (m), 2.20 (m)
22	35.0 CH_2_	1.75 (m, overlap)	38.3 CH_2_	2.13 (m), 1.93 (m)
23	29.2 CH_3_	1.03 (s)	206.3 CH	9.67 (s)
24	17.6 CH_3_	0.81 (s)	10.6 CH_3_	1.43 (s)
25	16.7 CH_3_	0.82 (s)	16.9 CH_3_	1.00 (s)
26	17.4 CH_3_	0.76 (s)	17.2 CH_3_	0.96 (s)
27	25.9 CH_3_	1.06 (s)	26.0 CH_3_	1.23 (s)
28	179.9 C	―	179.4 C	―
29	25.6 CH_3_	1.32 (s)	107.0 CH_2_	4.77 (s), 4.81 (s)

Recorded in pydine-*d_5_* at 600 MHz for ^1^H-NMR, 150 MHz for ^13^C-NMR.

The NOE correlations of H-2 with Me-24 (*δ*_H_ 0.81) and Me-25 (*δ*_H_ 0.82), and the large proton spin-coupling constant of H-3 (^3^*J*_H-2,H-3_ = 9.5 Hz) further supported that the hydroxyl groups at C-2 and C-3 were *α*- and *β*-orientation, respectively [5]. Therefore, **1** was unambiguously identified as 2*α*,3*β*,20*α*-trihydroxy-30-norolean-12-en-28-oic acid.

Compound **2** was obtained as a white amorphous powder with molecular formula C_29_H_42_O_5_ as determined by HRESIMS. The ^1^H- and ^13^C-NMR data (see Table 1) suggested that **2** is also a pentacyclic nortriterpenoid. Careful analysis of the ^1^H- and ^13^C-NMR spectra indicated that **2** closely resembled 2*α*,3*β*-dihydroxy-30-noroleana-12,20(29)-dien-28-oic acid [5], a literature reported noroleanane triterpenoid which was also obtained in this study as compound **4**. Except that the resonances for the methyl group at C-23 in **4** were replaced by signals [*δ*_H _9.67 (1H, s, H-23); *δ*_C_ 206.3 (C-23)] for an aldehyde group in **2**. These findings led to establish the structure of **2** as shown in Figure 1, which was well supported by the 2D NMR data. In the HMBC spectrum, the ^1^H−^13^C long-range correlations from *δ*_H_ 9.67 (1H, H-23) to *δ*_C_ 10.6 (C-24), and from *δ*_H_ 4.06 (1H, H-3) to *δ*_C_ 206.3 (C-23) and 10.6 (C-24) were observed, which confirmed the attachment of the aldehyde group at C-4 (Figure 3). The HMBC correlations from *δ*_H_ 4.24 (1H, H-2) to *δ*_C_ 76.9 (C-3), and from *δ*_H_ 4.06 (1H, H-3) to *δ*_C_ 67.9 (C-2), 56.5 (C-4), 206.3 (C-23) and 10.6 (C-24), and the large proton spin-coupling constant of H-3 (^3^*J*_H-2,H-3_ = 9.3 Hz) supported the *β*- and *α*-orientation of the hydroxyls at C-3 and C-2, respectively [5]. The HMBC correlations from H_2_-29 (*δ*_H_ 4.81, 4.77) to C-19 (*δ*_C_ 41.8) and C-21 (*δ*_C_ 30.2) confirmed the exocyclic double bond at C-20(29). The *α*-orientation of the aldehyde group at C-4 was evidenced by significant NOE correlations in the NOESY spectrum of H-23 (*δ*_H_ 9.67) with H-3 (*δ*_H_ 4.06) and H-5 (*δ*_H_ 1.64), and of H-2 with H_3_-24 (*δ*_H_ 1.43) and H_3_-25 (*δ*_H_ 1.00) (Figure 2). Therefore, compound **2** was determined as 2*α*,3*β*-dihydroxy-23-oxo-30-norolean-12,20(29)-dien-28-oic acid.

The four known compounds were identified as 3*β*-akebonoic acid (**3**) [16], 2*α*,3*β*-dihydroxy-30-noroleana-12,20(29)-dien-28-oic acid (**4**) [5], 3*α*-akebonoic acid (**5**) [16] and quinatic acid (**6**) [17] by comparison of their spectral data (^1^H and ^13^C-NMR and MS) to those reported in the literature. They were all reported here from the pericarps of *A. trifoliata* for the first time.

The *in vitro* antibacterial activity of the six isolated compounds against four Gram-positive bacteria [*St.* *aureus* (CMCC26003), *B.* *cereus* (CMCC63302), *B.* *subtilis* (CMCC63501) and *MRSA*] and three Gram-negative bacteria [*E.* *coli* (CMCC44102), *Sa.* *typhimurium* (CMCC44102) and *Sh.* *dysenteriae* (CMCC51252)] were evaluated using a microdilution titre assay as described in the Experimental section. As shown in Table 2, compound **3** displayed the best *in vitro* bacteriostatic activity against all the Gram positive bacteria assayed with MICs from 12.5 to 25 μg/mL. Compounds **4**, **5** and **6** showed bacteriostatic activity against *B. cereus* CMCC63302 and *B. subtilis* CMCC63501 with MICs of 50 and 25, of 25 and 50, and of 50 and 12.5 μg/mL, respectively. Neither compound showed antibacterial activity toward the three assayed Gram-negative bacteria strains (MICs > 200 μg/mL). It is noteworthy that compound **3** showed bacteriostatic activity against *MRSA* with MIC 25 μg/mL, which was much stronger than reference compound of Kanamycin sulfate (MIC 125 μg/mL). *MRSA*, firstly reported as a methicillin-resistant *Staphylococcus aureus* in the U.K. in 1961, is now a multidrug-resistant strain responsible for a rapidly increasing number of serious infectious diseases throughout the world, which is lacking of effective antimicrobial agents for the control and therapy for its infection.

**Table 2:** MIC values of compounds **1**–**6** in μg/mL against seven bacterial strains.

Bacteria	1	2	3	4	5	6	K	C
***S. aureus ***(CMCC26003)	>200	>200	25	200	>200	>200	1.9	50
***MRSA***	>200	>200	25	200	>200	>200	125	>200
***B. cereus ***(CMCC63302)	>200	>200	25	50	25	50	3.9	200
***B. subtilis*** (CMCC63501)	>200	>200	12.5	25	50	12.5	3.9	200
***E. coli ***(CMCC44102)	>200	>200	>200	>200	>200	>200	3.9	12.5
***S. typhimurium ***(CMCC44102)	>200	>200	>200	>200	>200	>200	3.9	12.5
***S. dysenteriae ***(CMCC51252)	>200	>200	>200	>200	>200	>200	3.9	12.5

K = kanamycin sulfate, C = cefradine.

These compounds were also tested for their *in vitro* cytotoxicity against three human tumor cell lines, A549 (human lung adenocarcinoma), HeLa (human cervical carcinoma) and HepG2 (human liver hepatocellular carcinoma), using the MTT method as described. The resulting IC_50_ values are displayed in Table 3. Compounds **4** and **5** showed interesting cytotoxicity against A549 and HeLa cell lines, with IC_50_ values 8.8 and 5.6 μM, respectively. Compound **3** also showed cytotoxic activity (IC_50_ 49.48, 28.63 and 52.89 μM) against the three tested tumor cell lines, but it was weaker than **4** and **5**. New compounds **1**–**2** and compound **6** did not exhibit cytotoxic activity (IC_50_ > 100 μM) in this bioassay. It could be deduced that the exocyclic double bond at C-20(29) might be an important active center for this type of nortriterpenoids to “maintain” their potential cytotoxicity, based on comparison of the structures and activities of **1** to **4**. Comparison of the chemical structures of **3** and **4** *versus* **5** indicated that the *α*-orientation of the hydroxyl group at C-3 could strengthen the cytotoxic activity of this type of nortriterpenoids, while the *α*-hydroxyl group at C-2 seems not necessary. Moreover, a negative effect on the cytotoxicity was evident when Me-23 was oxidized (*i.e.*, replaced by an -CHO group), as supported by analyzing the structure-active relationship of **2** and **4**.

**Table 3:** Cytotoxicity of compounds **1**–**6** (IC_50_, µM).

Compounds	A549	HeLa	HepG2
**1**	>100	>100	>100
**2**	>100	>100	>100
**3**	49.48 ± 8.64	28.63 ± 7.41	52.89 ± 5.28
**4**	8.770 ± 0.59	16.33 ± 0.12	14.28 ± 0.49
**5**	10.59 ± 0.69	5.61 ± 0.00	10.39 ± 1.17
**6**	>100	>100	>100
Adriamycin	0.69 ± 0.07	0.47 ± 0.06	1.22 ± 0.02

Values represent mean ± SD (n = 3) based on three individual experiments.

Compounds **1**, **2**, **5** and **6** were further tested for their *α*-glucosidase inhibitory activity. The results are listed in Table 4, with acarbose used as a reference compound. Compound **5** showed the best *α*-glucosidase inhibitory activity with IC_50_ value 0.035 mM, which was about twelve-fold stronger than acarbose (IC_50_ 0.409 mM). Compound **6** showed significant *α*-glucosidase inhibitory activity with IC_50_ value (0.10 mM) about four-fold stronger than the reference compound. Though the *α*-glucosidase inhibitory activities of **1** and **2** (IC_50_ 0.367 and 0.220 mM, respectively) were inferior to **5** and **6**, they were still more potent than acarbose. These results indicated that 30-noroleanane triterpenes in the pericarps of *A. trifoliata* were effective *α*-glucosidase inhibitors promising to be developed as effective and safe hypoglycemic agents for diabetes chemotherapy [18].

**Table 4:** *α*-Glucosidase inhibitory activity of compounds **1**, **2**, **5** and **6**.

Compounds	IC_50_ (mM)
**1**	0.367 ± 0.003
**2**	0.220 ± 0.004
**5**	0.035 ± 0.002
**6**	0.100 ± 0.001
Acarbose	0.409 ± 0.006

Values represent mean ± SD (n = 3) based on three individual experiments.

## 3. Experimental

### 3.1. General Information

Optical rotation were measured on a Perkin-Elmer 341 polarimeter (Perkin- Elmer, Waltham, MA, USA) with MeOH as solvent at the wavelength of 589 nm and 20 °C to gain their specific optical rotation [α] values after calculation. Nuclear magnetic resonance (NMR) spectra were recorded on a Bruker Advance 600 NMR spectrometer (Bruker Biospin corporation, Billerica, MA, USA), Bruker advance 500M NMR spectrometer (Bruker) and a Bruker DRX-400 NMR spectrometer (Bruker Biospin, Rheistetten, Germany) with the solvent residual peaks of *δ*_H_ 7.22, 7.58, 8.74 and *δ*_C_ 123.4, 135.4, 149.7 for pyridine-*d_5_*, and *δ*_H_ 2.50 and *δ*_C_ 39.5 for DMSO-*d_6_*. HR-ESI-MS mass spectra were obtained on a Bruker maXis instrument (Bruker Daltonik GmbH, Bremen, Germany) in a positive ion mode after direct injection of the test solutions. ESI-MS data were obtained using a MDS SCIEX API 2000 LC/MS/MS system (Applied Biosystems, Foster City, CA, USA) in both positive and negative ion modes in the range of *m/z* 50–1000 after the test solutions were directly injected into the ESI source by a syringe pump. Preparative HPLC was carried out on a CXTH P3000 HPLC pump and a UV 3000 UV-Vis Detector with a Fuji-C18 column (10 um–100 A, ChuangXinTongHeng Science And Technology Co., Ltd, Beijing, China); the Performance of MPLC (medium pressure liquid chromatography) is a CXTH P3000 HPLC pump, a UV 3000 UV-Vis Detector and a C18 column (400 × 25 mM i.d, 50 μM, YMC Co. Ltd., Kyoto, Japan). 

Column chromatography (CC) was performed with silica gel (80–100 and 200–300 mesh, Qingdao Haiyang Chemical Co., Qingdao, China), YMC ODS-A (50 μm, YMC Co. Ltd., Kyoto, Japan), Sephadex LH-20 (Pharmacia Fine Chemical Co. Ltd., Uppsala, Sweden), MCI gel CHP 20P (75–150 μM, Mitsubishi Chemical Corp., Tokyo, Japan). Analytical grade petroleum ether (b.p. 60–90 °C), methanol, ethyl acetate, chloroform, *n*-butanol, acetone were purchased from Tianjin Fuyu Fine Chemical Industry Co. (Tianjin, China); HPLC grade methanol was purchased from J&K Chemical Ltd. (Beijing, China); Fraction were monitored by precoated HSGF_254_ TLC (Yantai Jiangyou Silica Gel Co. Ltd, Yantai, China), and spot detection was performed under fluorescent light (λ = 254 nm), and then spraying 10% H_2_SO_4_ in ethanol, followed by heating. Kanamycin sulfate, resazurin, pyridine-*d_5_*, DMSO-*d_6_*, 3-(4,5-dimethylthiazol-2-yl)-2,5-diphenyltetrazolium bromide (MTT) and *α*-glucosidase were purchased from Sigma Chemical Co. (Sigma-Aldrich, St. Louis, MO, USA). Roswell Park Memorial Institute (RPMI)-1640 medium and fetal calf serum were purchased from Gibco BRL (Gaithersburg, MD, USA). Adriamycin was obtained from Pfizer Italia SRL (Roma, Italy). *p*-Nitrophenyl-*α*-d-glucopyranoside (PNPG) and acarbose were obtained from Tokyo Chemical Industry Co., Ltd. (Tokyo, Japan).

### 3.2. Plant Materials

The pericarps of *Akebia trifoliata* were collected in September 2009, at Liye of Longshan, Hunan Province, China, identified by Prof. Fu-Wu Xing at South China Botanical Garden, the Chinese Academy of Sciences (CAS). A voucher specimen (No. 20090920) was deposited at the Laboratory of Bioorganic Chemistry of the South China Botanical Garden, Chinese Academy of Sciences.

### 3.3. Extraction and Isolation

Air-dried pericarps of *Akebia trifoliata* (3 kg) were powdered and extracted three times (3 days each) with 95% EtOH (9 L × 3) at room temperature (25–32 °C). The concentration of the solution under vacuum gave a reddish solid. Then, the resulting residue was suspended in H_2_O (1.5 L) and successively partitioned with petroleum ether (1.5 L × 3) and ethyl acetate (1.5 L × 3) to afford petroleum ether-soluble (40.0 g) and EtOAc-soluble (180 g) fractions after condensation to dryness *in vacuo*. The petroleum ether-soluble fraction was subjected to silica gel column chromatography (1000 × 105 mM i.d.) using a gradient system of petroleum ether-acetone (100:0, 20:1, 10:1, 5:1, 2:1, 1:1, 0:100, *v/v*, each 1.5 L) to give nine fractions (E_1_-E_9_) after pooled according to their TLC profiles. E_3_ (0.45 g) was repeatedly chromatographied on Sephadex LH-20 column (1550 × 13.4 mM i.d.) eluted with acetone to yield compound 5 (6.5 mg). E_4_ (1.7 g) was purified on silica gel column chromatography (1000 × 105 mM i.d.) using petroleum ether-acetone (10:1, 9:1, 8:1, 7:1, 6:1, *v/v*, each 250 mL) as elution system, and then further purified by using MPLC eluted with MeOH-H_2_O (95:5, *v/v*) to yield compound **3** (2.2 mg). E_7_ (1.8 g) and E_8_ (2 g) were passed through a MCI gel column (200 × 40 mM i.d.) for depigmentation. The resultant methanolic eluate (0.9 g) of E_7_ was sequentially separated by MPLC eluted with a gradient of methanol in water (75:25, 80:20, 85:15, 90:10, 100:0, *v/v*, each 70 mL), and by Sephadex LH-20 column (1550 × 13.4 mM i.d.) chromatography eluted with MeOH, and silica gel CC using CHCl_3_-MeOH (98:2, *v/v*) elution to obtain compounds **2** (4 mg) and compound **4** (2.0 mg). The resultant methanolic eluate (1.1 g) of E_8_ was sequentially separated by MPLC eluted with a gradient of methanol in water (60:0, 70:30, 80:20, 90:10, 100:0, *v/v*, each 100 mL) to give eight fractions (E_8-1_-E_8-8_). The EtOAc-soluble fraction was subjected to silica gel CC (1000 × 105 mM i.d.) using a gradient of CHCl_3_-MeOH (97:3, 90:10, 85:15, 70:30, 60:40, 0:100, *v/v*, each 3 L) to give ten fractions (F_1_-F_6_). Fraction F_3 _(6.8 g), obtained by elution CHCl_3_-MeOH (85:15, *v/v*), was further subjected to silica gel CC (800 × 50 mM i.d.) and successively eluted with CHCl_3_-MeOH (98:2, 95:5, 90:10, *v/v*, each 0.5 L) to yield six sub-fractions (F_5-1_–F_5-6_), Sub-fraction F_5-3 _(1.6 g) was separated by MPLC using MeOH-H_2_O (60:40, 70:30, 80:20, 90:10, 100:0, *v/v*, each 350 mL) at a flow rate of 10 mL/min, and further purified by Sephadex LH-20 column (1550 × 13.4 mM i.d.) chromatography eluted with MeOH to obtain compound **6** (9 mg). Fraction F_5_ (20.5 g), obtained by elution CHCl_3_-MeOH (60:40, *v/v*), was further subjected to silica gel CC (1000 × 105 mM i.d.), and successively eluted with CHCl_3_-MeOH (90:10, 80:20, 70:30, 60:40, *v/v*, each 1.5 L) to obtain six sub-fractions (F_5-1_-F_5-6_). F_5-3_ (10 g) was separated by MPLC, and eluted with MeOH-H_2_O (70:30, *v/v*) to yield compound **1** (5 mg).

*2α,3β,20α-Trihydroxy-30-norolean-12-en-28-oic acid* (**1**). White amorphous powder. 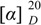 +106.4 (*c* 0.70, MeOH). IR (KBr) *ν*_max_ 3428, 2939, 2873, 1699, 1459, 1380, 1286, 1450, 1116, 1047 cm^−1^. ESI-MS (+) *m*/*z*: 497 [M+Na]+, 971 [2M+Na]+; ESI-MS (−) *m*/*z*: 473 [M−H]^–^, 947 [2M−H]^–^;. HR-ESI-MS *m*/*z*: 497.3235 [M+Na]+ (calcd for C29H46NaO5, 497.3237). For ^1^H-NMR (600 MHz, C_5_D_5_N) and ^13^C-NMR (150 MHz, C_5_D_5_N) data, see Table 1.

*2α,3β-Dihydroxy-23-oxo-30-norolean-12,20(29)-dien-28-oic acid* (**2**). White amorphous powder. 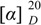 +190.0 (*c* 0.17, MeOH); IR (KBr) *ν*_max_ 3413, 2935, 2861, 1720, 1689, 1456, 1384, 1292, 1220, 1054 cm^−1^. ESI-MS (+) *m*/*z*: 493 [M+Na]^+^, ESI-MS (−) *m*/*z*: 469 [M−H]^–^. HR-ESI-MS *m*/*z*: 493.2922 [M+Na]^+^ (calcd for C_29_H_42_NaO_5_, 493.2924). For ^1^H-NMR (600 MHz, C_5_D_5_N) and ^13^C-NMR (150 MHz, C_5_D_5_N) data, see Table 1.

### 3.4. Antibacterial Assay

The bacteriostatic activity of compounds **1**–**6** were monitored according to the method of Rahman with slight modifications [19]. Briefly, indicator solution (100 μL, resazurin in sterile water, 100 μg/mL) was first placed into each of the sterility control wells (11th column) on the 96 well plates, and indicator solution (about 7.5 mL, 100 μg/mL) was mixed with test organism (5 mL, 10^6^ cfu/mL, OD_600_ = 0.07) followed by transferring (100 μL, each) to growth control wells (12th column) and all test wells (1–10th column). Then, each of 100 μL of the test samples in beef extract peptone medium, the positive control solution were prepared by adding kanamycin sulfate and cefradine instead of the samples and the negative control solution (3% DMSO of beef extract peptone medium) were applied to the wells in the 1st column of the plates. Once all samples and controls were properly applied to the 1st column of wells on the plate, half of the homogenized content (100 μL) from these wells was then parallel transferred to the 2nd column of wells, and each subsequent well was treated similarly (doubling dilution) up to the 10th column, followed by discarding the last 100 μL aliquot. Finally, the plates were incubated at 37 °C for 5–6 h until the color of growth control change to pink. The lowest concentration for each test compound at which color change occurred was recorded as the MIC value of the test compound. The Gram-(+) bacteria, *Staphyloccocus aureus* (CMCC26003), *Bacillus cereus* (CMCC63302) and *Bacillus subtilis* (CMCC63501), and Gram-(‒) bacteria *Escherichia coli* (CMCC44102), *Salmonella typhimurium* (CMCC44102) and *Shigella dysenteriae* (CMCC51252) were obtained from the Guangdong Institute of Microbiology (Guangzhou, China). The multi-drug resistant *Staphyloccocus aureus* (*MRSA*) was kindly provided by Dr. Wei, X.Y. (South China Botanical Garden).

### 3.5. Cytotoxic Assay

Compounds **1**−**6** were evaluated for their cytotoxicity against three human cancer cell lines, human lung adenocarcinoma (A549), human cervical carcinoma (HeLa) and human liver hepatocellular carcinoma (HepG2). The three tumor cell lines were generously provided by Kunming Institute of Zoology, Chinese Academy of Sciences. The cytotoxic activities of the tested compounds were assayed according to the MTT method by using 96 well plates [20]. Briefly, the cells were cultured in RPMI-1640 medium, supplemented with 10% fetal bovine serum in a humidified atmosphere with 5% CO_2_ at 37 °C. One hundred μL of adherent cells at the density of 5 × 10^4^ cell/mL was seeded into each well of 96-well cell culture plates and incubated in 5% CO_2_ at 37 °C for 24 h to form a monolayer on the flat bottoms. Then, removed the supernatant per well and subsequently added with 100 μL of fresh medium and 100 μL of medium containing a test compound. The plate was then incubated in 5% CO_2_at 37 °C. After 72 h, 20 μL of 5 mg/mL MTT in DMSO was added into each well and incubated for 4 h. The supernatant per well was carefully removed and 150 μL of DMSO was added. The plate was then vortex shaken for 15 min to dissolve blue formazan crystals. The optical density (OD) of each well was measured on a Genois microplate reader (Tecan GENios, Männedorf, Switzerland) at the wavelength of 570 nm. All experiments were performed in triplicate and adriamycin was used as a positive control. In each experiment, each of the tumor cell lines was exposed to the test compound at concentrations of 50, 25, 12.5, 6.25, 3.125, 1.5625 µg/mL. The inhibitory rate of cell growth was calculated according to the following formula: Inhibition rate (%) = (OD_control_− OD_treated_)/OD_control_× 100%. IC_50_ values were calculated by SPSS 16.0 statistic software. The values were based on three individual experiments and expressed as means ± standard deviation (SD).

### 3.6. α-Glucosidase Inhibition Assay

The *a*-glucosidase inhibitory activity of **1**, **2**, **5** and **6** were determined spectrophotometrically in a 96-well microtiter plate based on *p*-nitrophenyl-*α*-d-glucopyranoside (PNPG) as a substrate following the method described in the literature with slight modifications [21,22]. In brief, *α-*glucosidase (20 μL, 0.8 U/mL) and various concentrations (500, 250, 125, 62.5, 31.25, 15.625 µg/mL) of tested compounds (120 μL) in 67 mM phosphate buffer (pH 6.8) were mixed at room temperature for 10 min. Reactions were initiated by addition of 5.0 mM PNPG (20 μL). The reaction mixture was incubated for 15 min at 37 °C in a final volume of 160 μL. Then, 0.2 M Na_2_CO_3_ (80 μL) was added to the incubation solution to stop the reaction. The activities were detected in a 96-well plate, and the absorbance was determined at 405 nm (for *p*-nitrophenol). The negative blank was set by adding phosphate buffer instead of the sample via the same way as the test. Acarbose was utilized as positive control. The blank was set by adding phosphate buffer instead of the *α-*glucosidase using the same method. Inhibition rate (%) = [(OD_negative control_− OD_blank_) − (OD_test _− OD_test blank_)]/(OD_negative blank_− OD_blank_) × 100%. IC_50_ values of the samples were calculated using the Microsoft Office Excel soft.

## 4. Conclusions

Two new 30-noroleanane triterpenes **1**–**2** were isolated from the pericarps of *A. trifoliata*, along with four known ones **3**–**6**. Their structures were identified by spectroscopic means, including NMR and HRESIMS. All the compounds were isolated from the pericarps of *A. trifoliata* for the first time. Bioassays showed that compounds **3**–**6** were selectively active against four tested Gram-(+) bacteria, especially **3** toward *MRSA* with a MIC value about five-fold stronger than the reference compound kanamycin. Compounds **4** and **5** were found to show moderate cytotoxic activity against human tumor A549 and HeLa cell lines. Compounds **1**, **2**, **5** and **6** were all revealed to show significant *in vitro* *α*-glucosidase inhibitory activities which were more potent than reference compound acarbose. These findings indicate that the pericarps of *A. trifoliata* is rich in 30-noroleanane triterpenoids which are promising for exploitation as functional food ingredients or to be developed as effective and safe agents to contribute to the control and therapy of human diseases.

## Supplementary Materials

Supplementary materials can be accessed at: http://www.mdpi.com/1420-3049/19/4/4301/s1.
