# Supplementary material for: Bioactive 30-Noroleanane Triterpenes from the Pericarps of Akebia trifoliata
Source: Molecules. 2014 Apr 4;19(4):4301–12. doi: 10.3390/molecules19044301 (PMC6271860; doi:10.3390/molecules19044301)

ESIMS(+) of Compound **1**

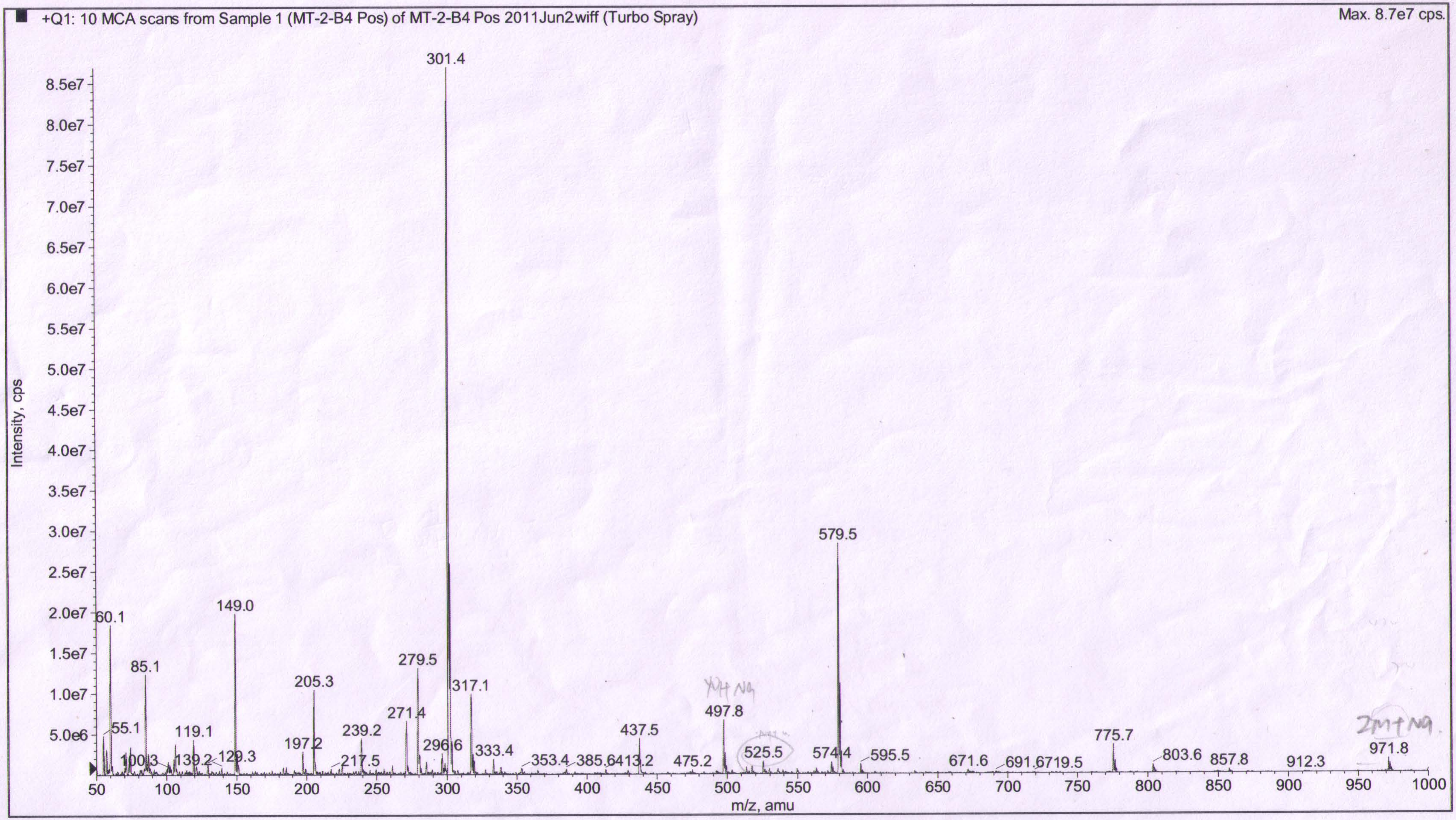

ESIMS(-) of Compound 1

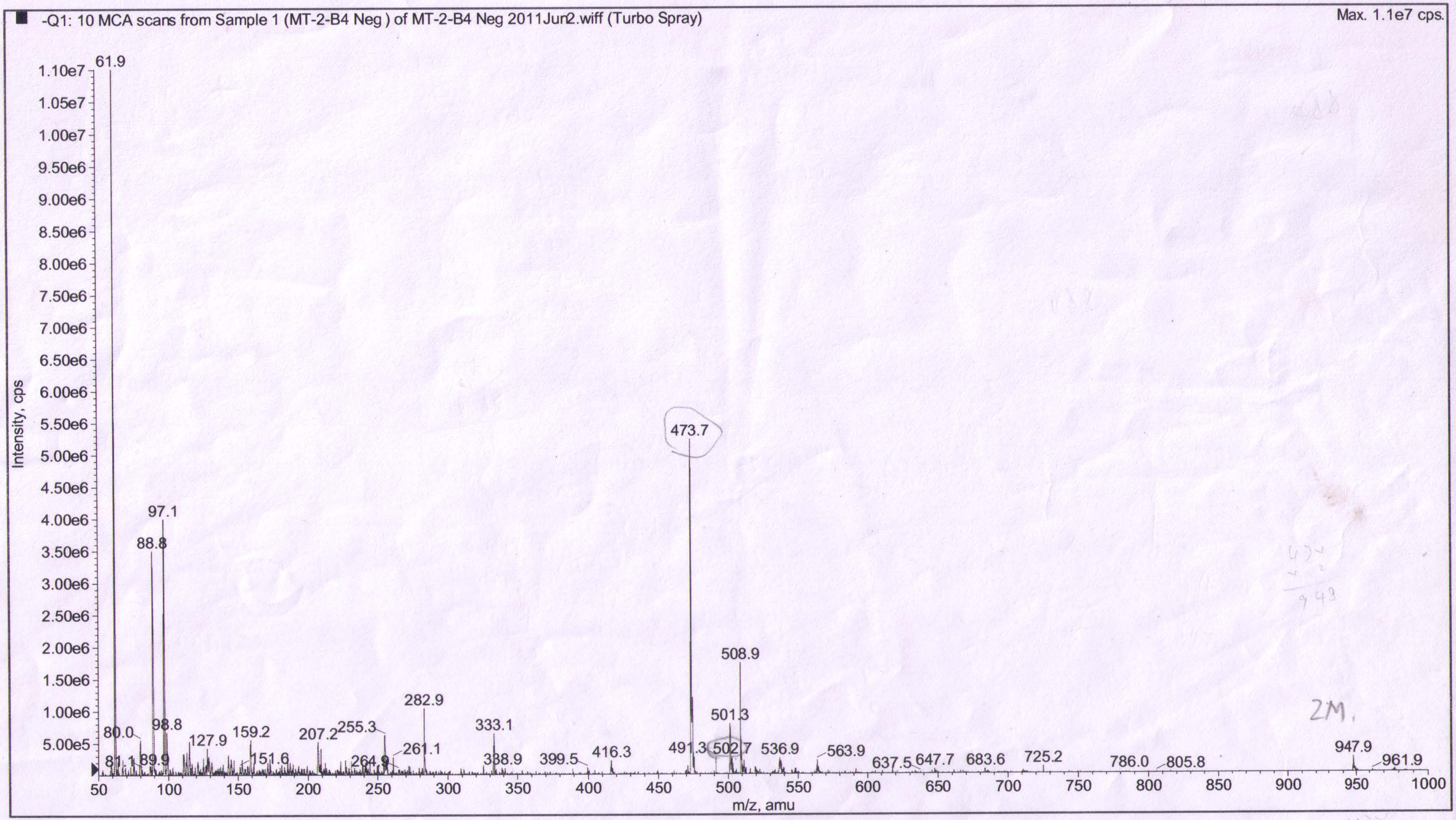

HRESIMS of Compound **1**

Mass Spectrum SmartFormula Report

Analysis Info

Analysis Name	D:\Data\MS\data\201211\wangjing-MT-2-B4 pos.d	Acquisition Date	11/21/2012 11:20:44 AM
Method	POS_100-2000_Dirrect Infusion.m	Operator	SCSIO
Sample Name		Instrument / Ser#	maXis 29
Comment			

Acquisition Parameter

Source Type	ESI	Ion Polarity	Positive	Set Nebulizer	0.3 Bar
Focus	Not active	Set Capillary	3500 V	Set Dry Heater	180 °C
Scan Begin	100 m/z	Set End Plate Offset	-500 V	Set Dry Gas	4.0 l/min
Scan End	2000 m/z	Set Collision Cell RF	2000.0 Vpp	Set Divert Valve	Waste

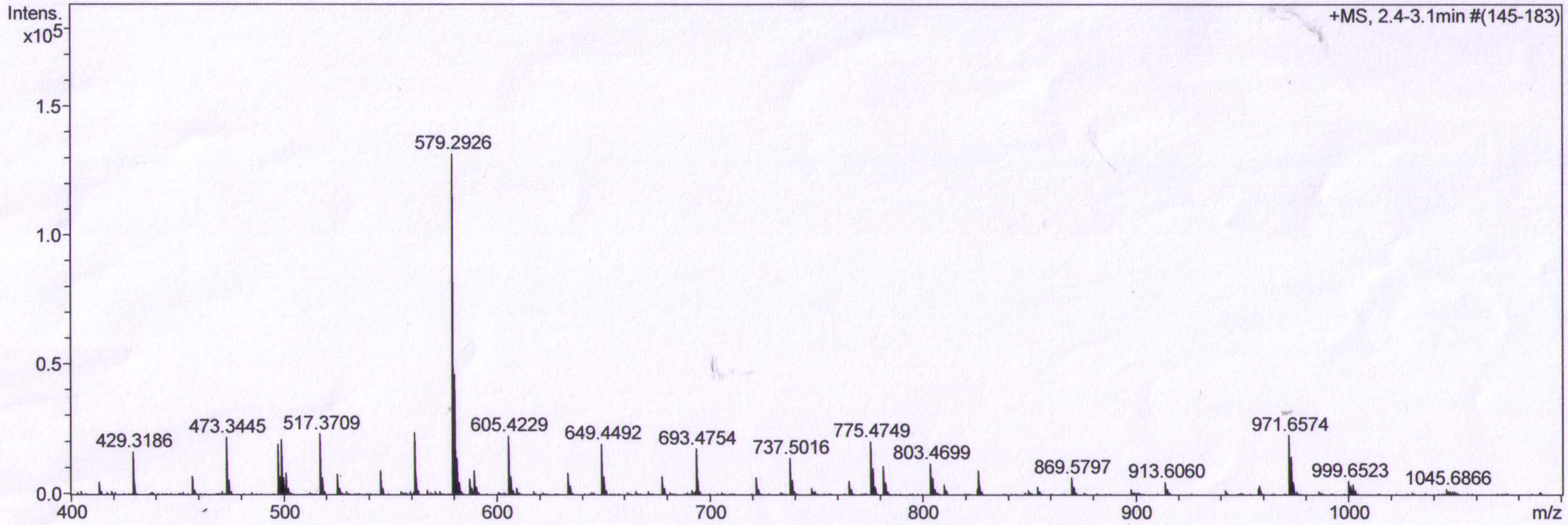

Meas. m/z	#	Formula	Score	m/z	err [mDa]	err [ppm]	mSigma	rdb	e⁻ Conf	N-Rule
497.3235	1	C 29 H 46 Na O 5	100.00	497.3237	0.3	0.6	497.5	6.5	even	ok
971.6574	1	C 58 H 92 Na O 10	100.00	971.6583	0.9	0.9	6.3	12.5	even	ok

^{1}HNMR spectrum of compound **1**

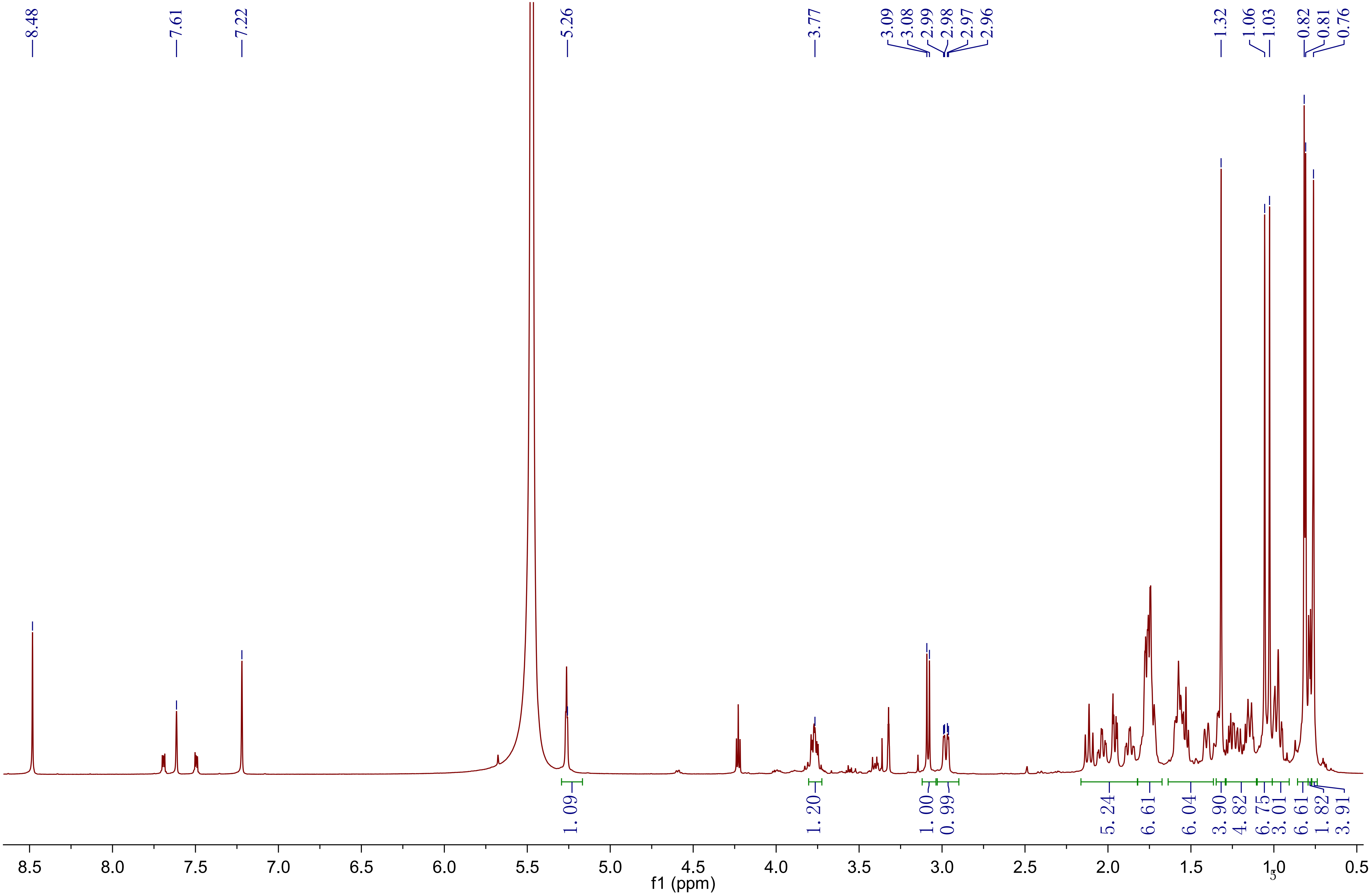

^{13}CNMR and DEPT spectra of compound **1**

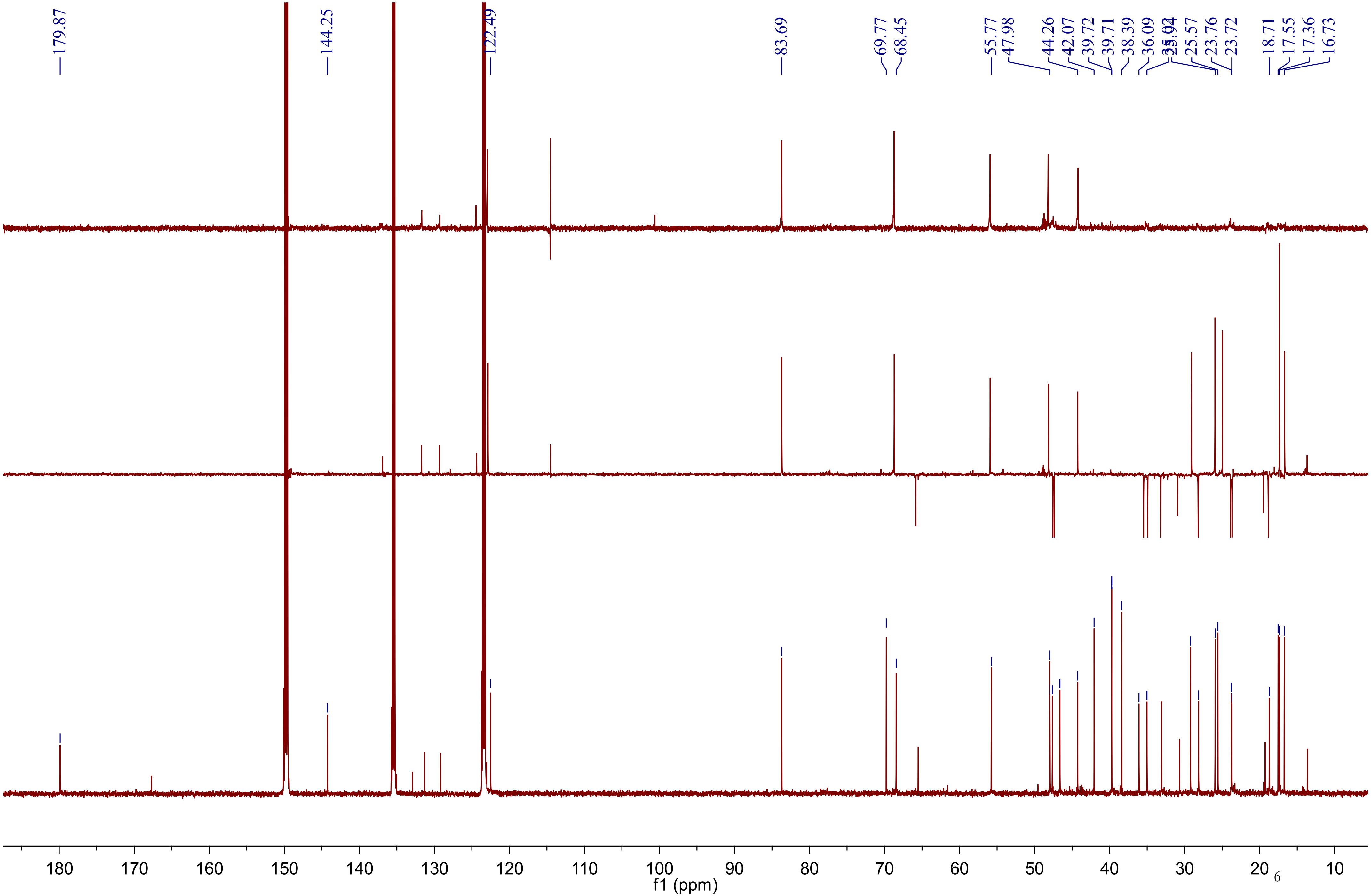

HSQC spectrum of compound 1

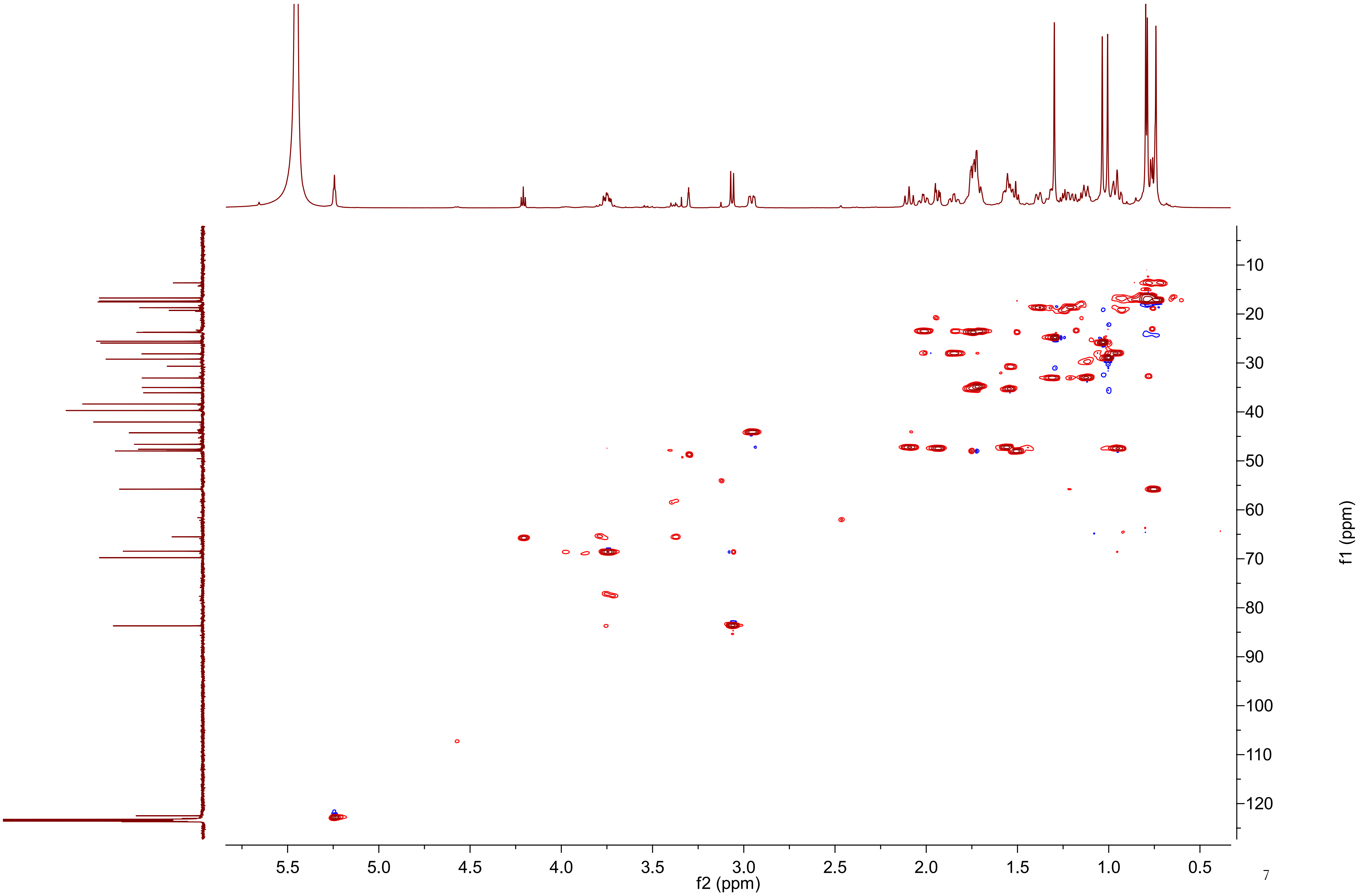

HMBC spectrum of compound 1

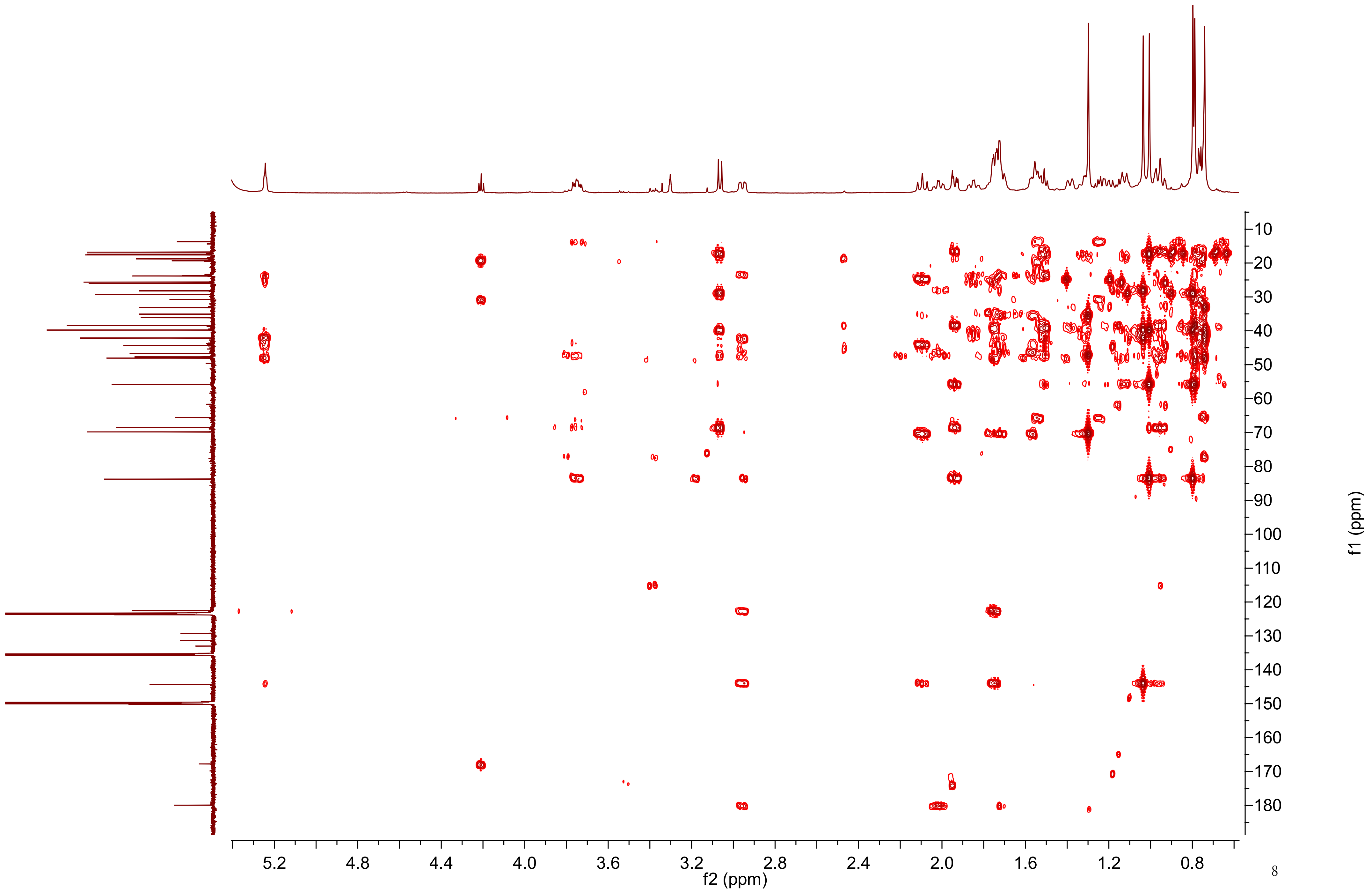

^{1}H-^{1}H COSY spectrum of compound 1

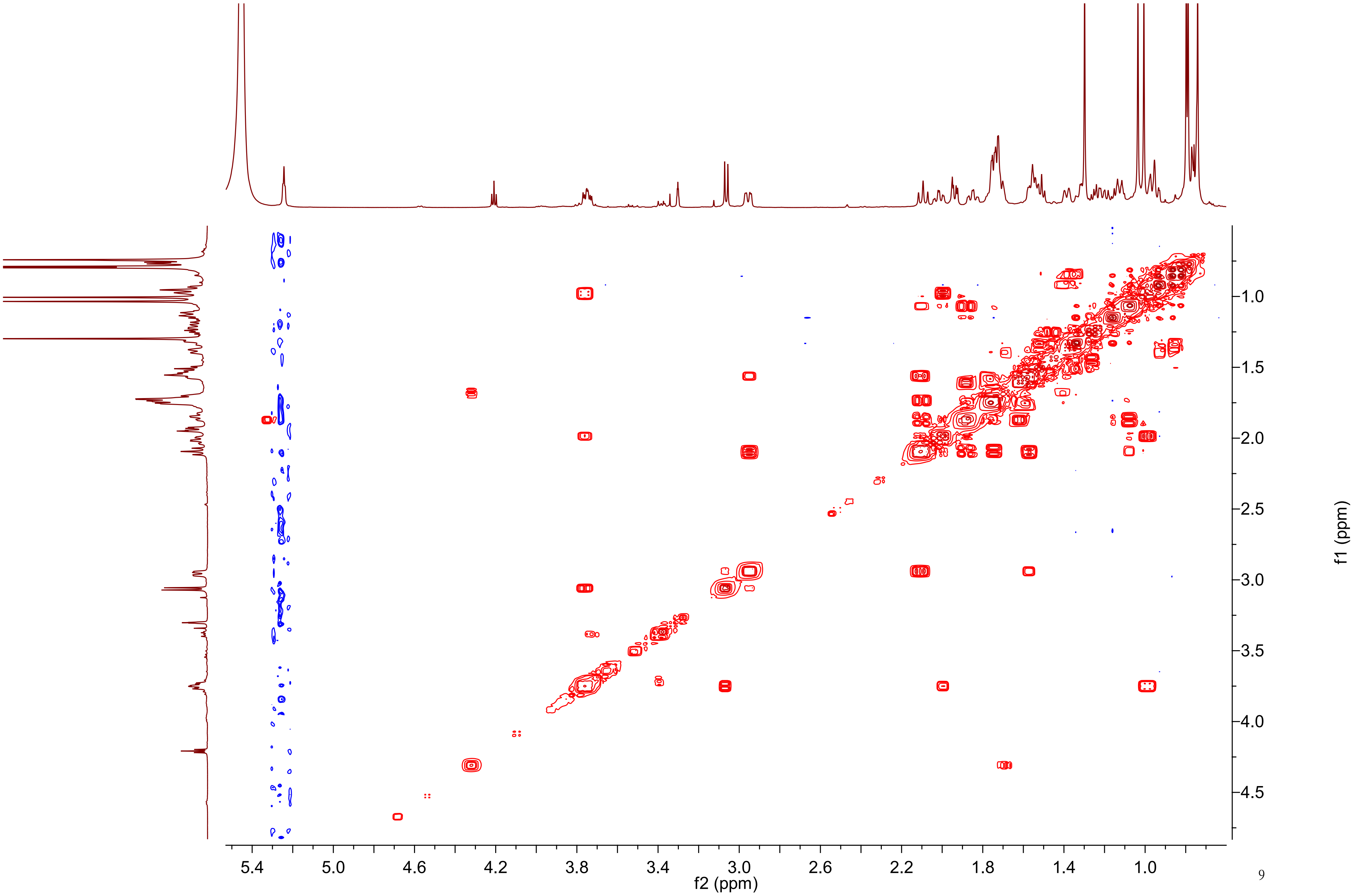

NOESY spectrum of compound 1

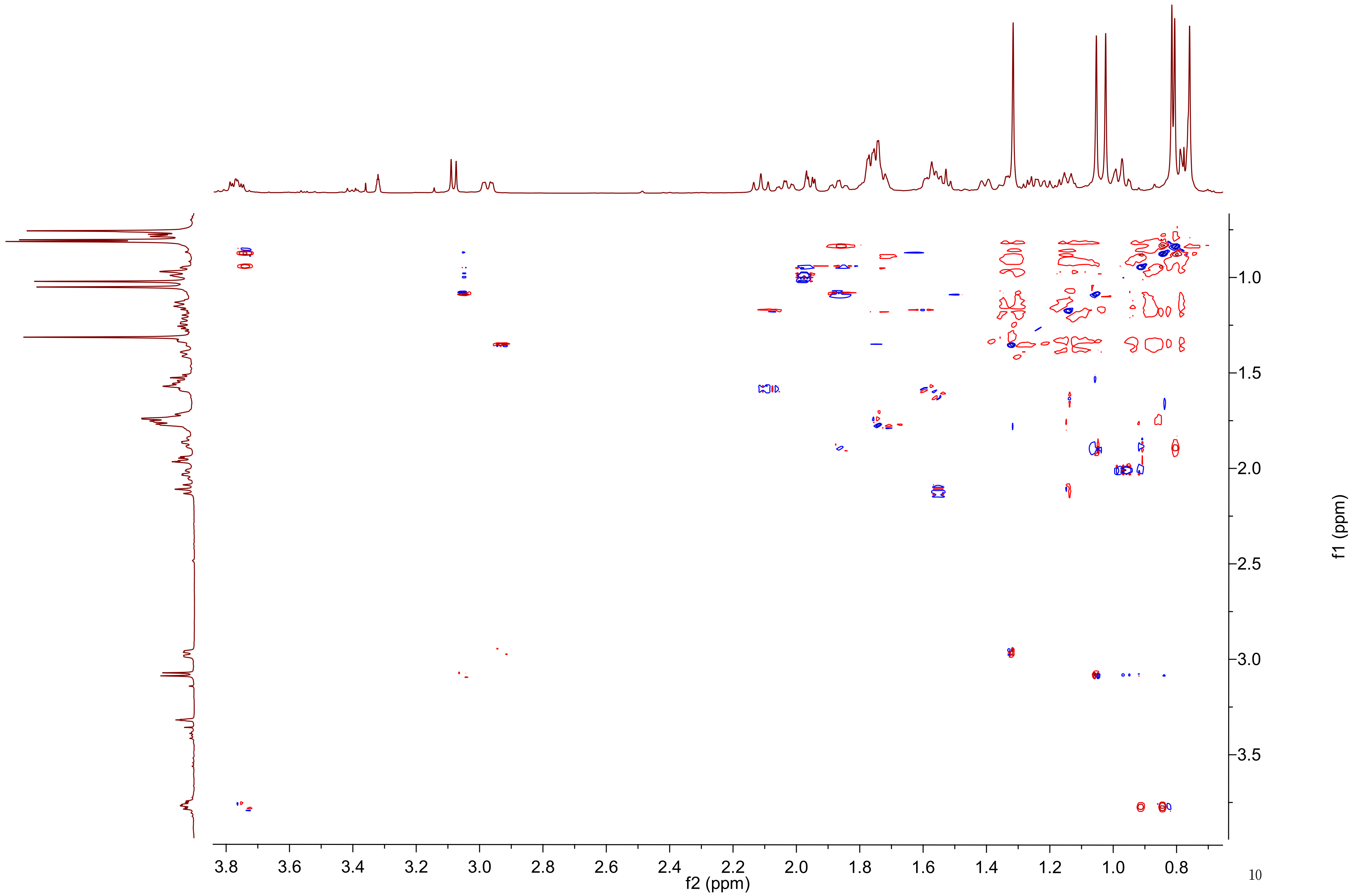

ESIMS(+) of Compound 2

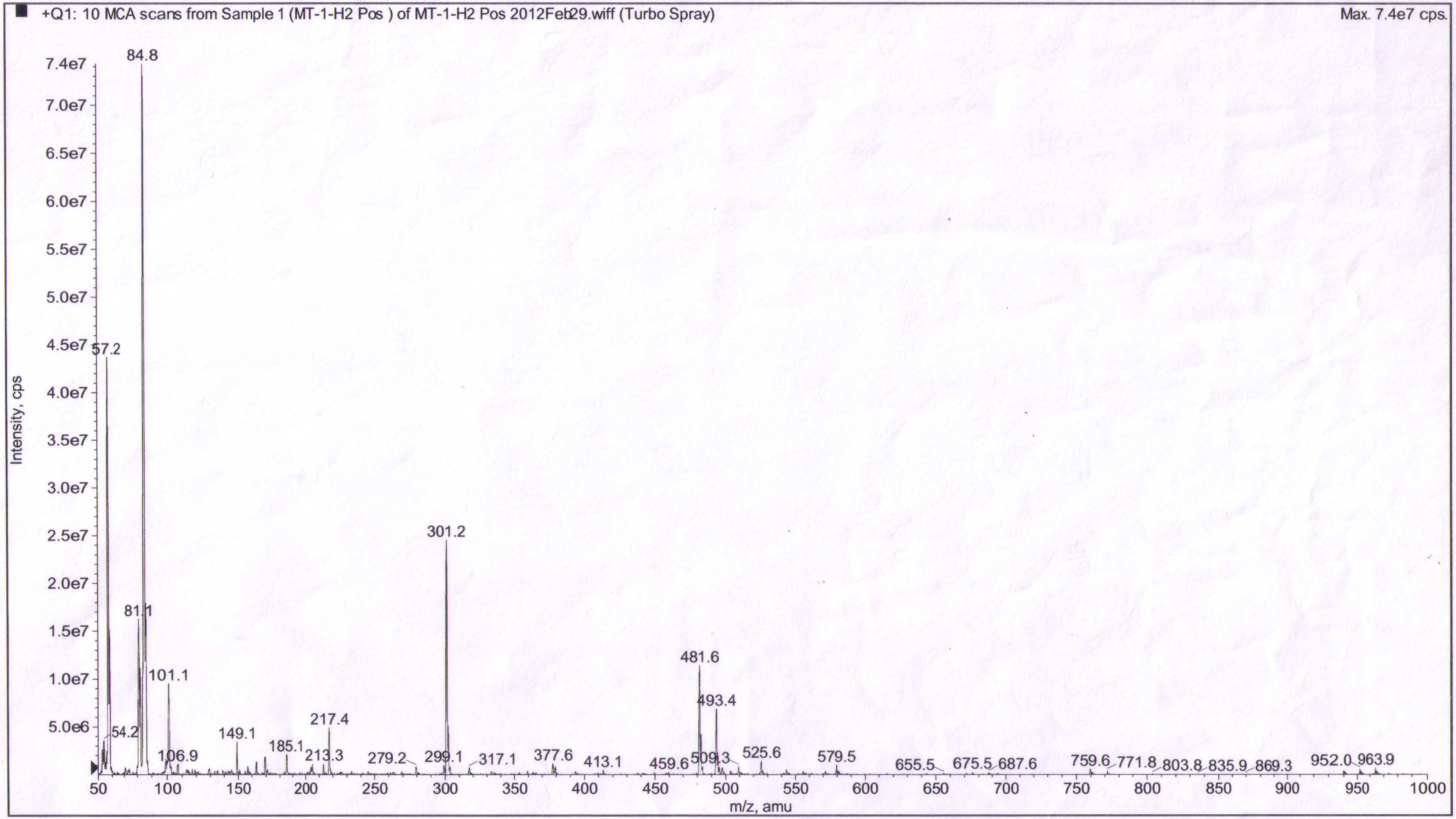

ESIMS(-) of Compound **2**

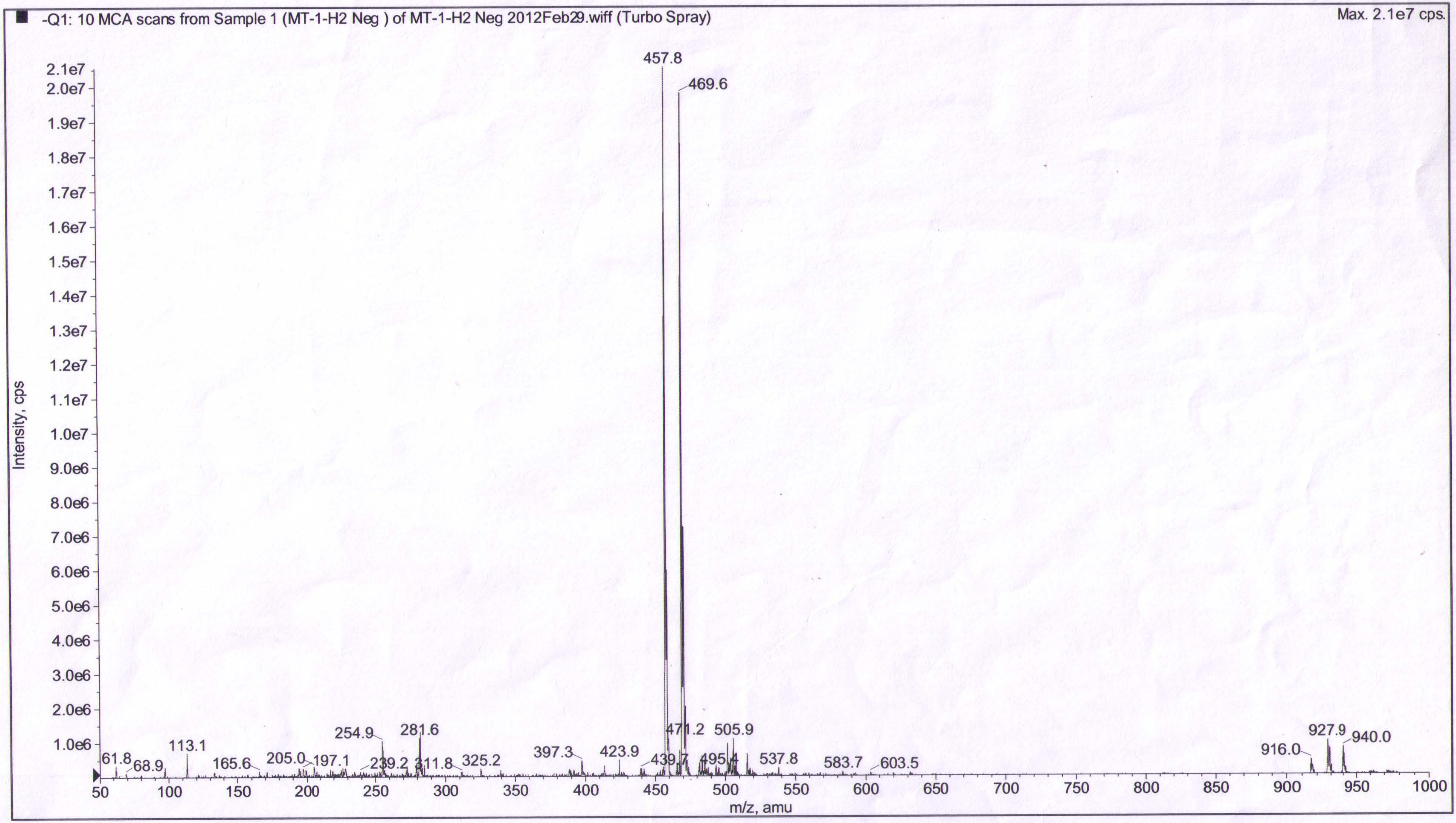

HRESIMS of Compound **2**

Mass Spectrum SmartFormula Report

Analysis Info

Analysis Name	D:\Data\MS\data\201211\wangjing-MT-1-H2 pos.d	Acquisition Date	11/21/2012 11:09:43 AM
Method	POS_100-2000_Dirrect Infusion.m	Operator	SCSIO
Sample Name		Instrument / Ser#	maXis 29
Comment			

Acquisition Parameter

Source Type	ESI	Ion Polarity	Positive	Set Nebulizer	0.3 Bar
Focus	Not active	Set Capillary	3500 V	Set Dry Heater	180 °C
Scan Begin	100 m/z	Set End Plate Offset	-500 V	Set Dry Gas	4.0 l/min
Scan End	2000 m/z	Set Collision Cell RF	2000.0 Vpp	Set Divert Valve	Waste

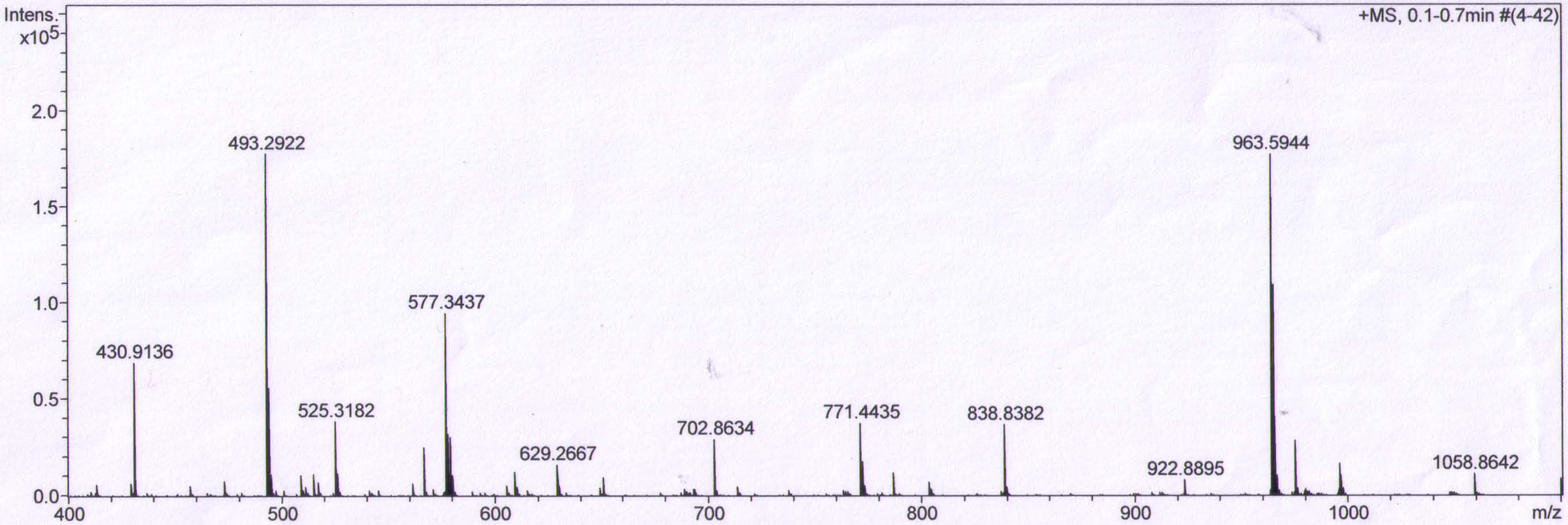

Meas. m/z	#	Formula	Score	m/z	err [mDa]	err [ppm]	mSigma	rdb	e⁻ Conf	N-Rule
493.2922	1	C 29 H 42 Na O 5	100.00	493.2924	0.2	0.5	3.2	8.5	even	ok
963.5944	1	C 58 H 84 Na O 10	100.00	963.5957	1.3	1.4	12.6	16.5	even	ok

Bruker Compass DataAnalysis 4.0 printed: 11/21/2012 11:29:08 AM Page 1 of 1

^{1}HNMR spectrum of compound **2**

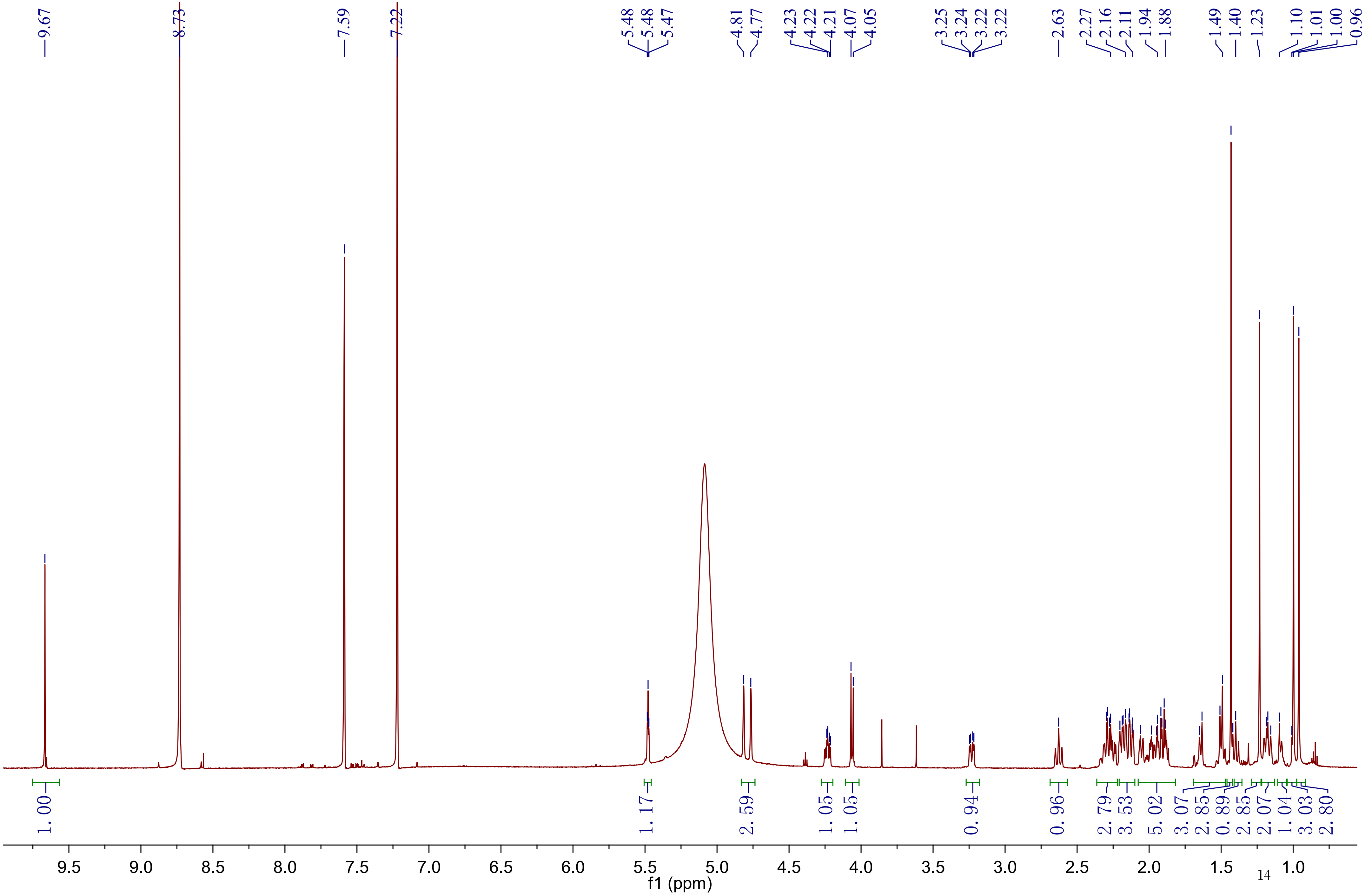

^{13}CNMR and DEPT spectra of compound **2**

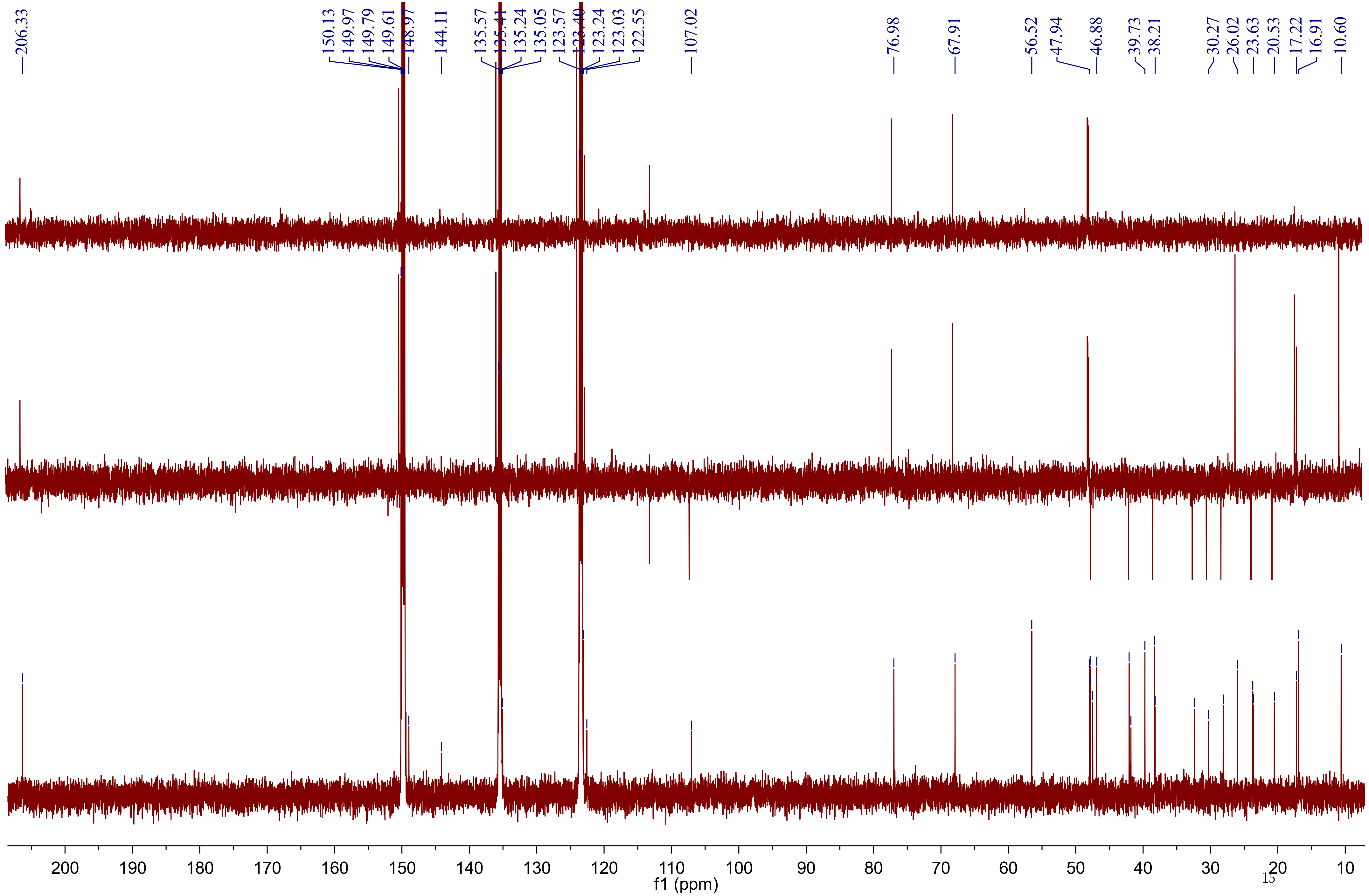

HSQC spectrum of compound **2**

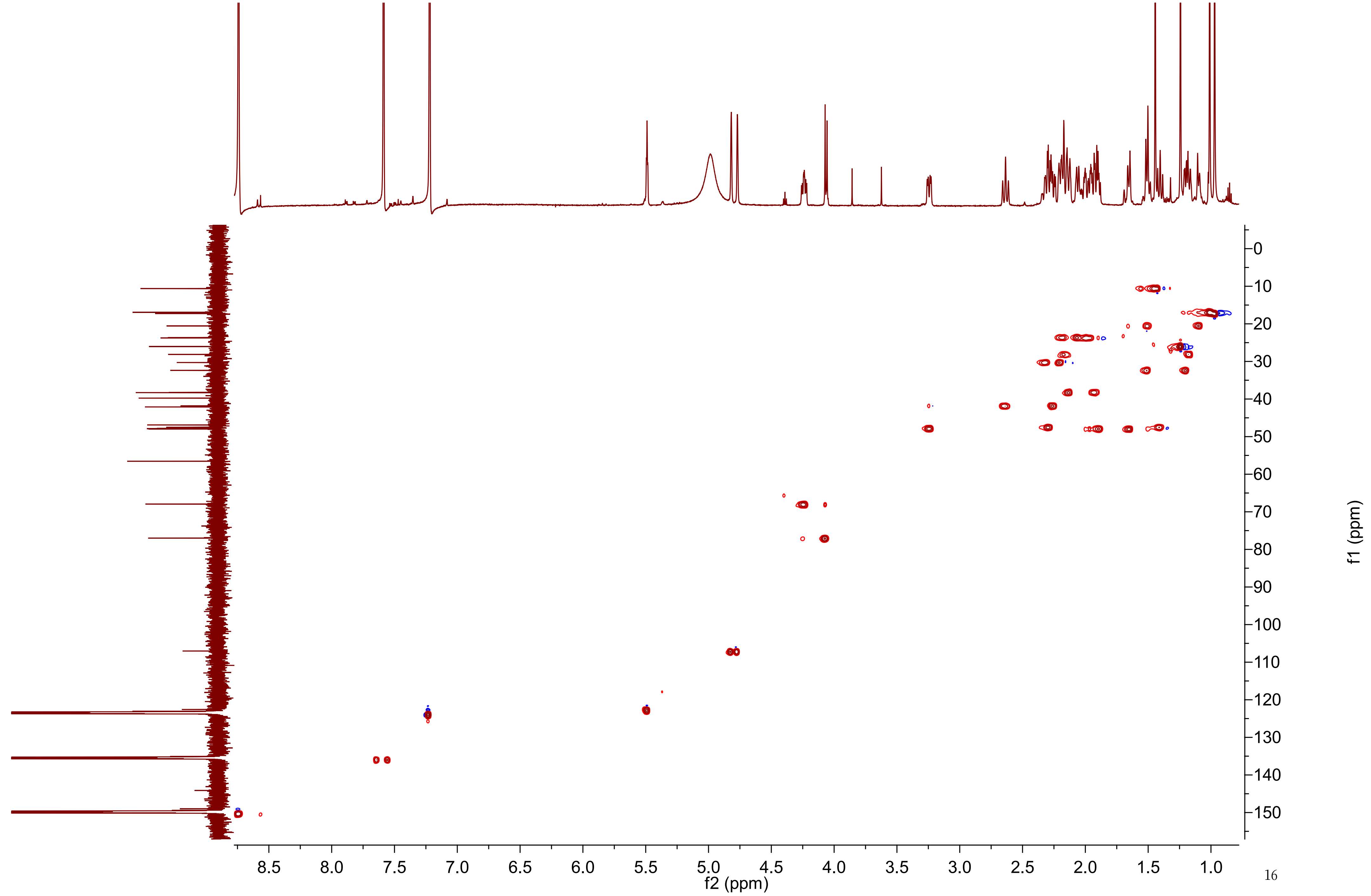

HMBC spectrum of compound 2

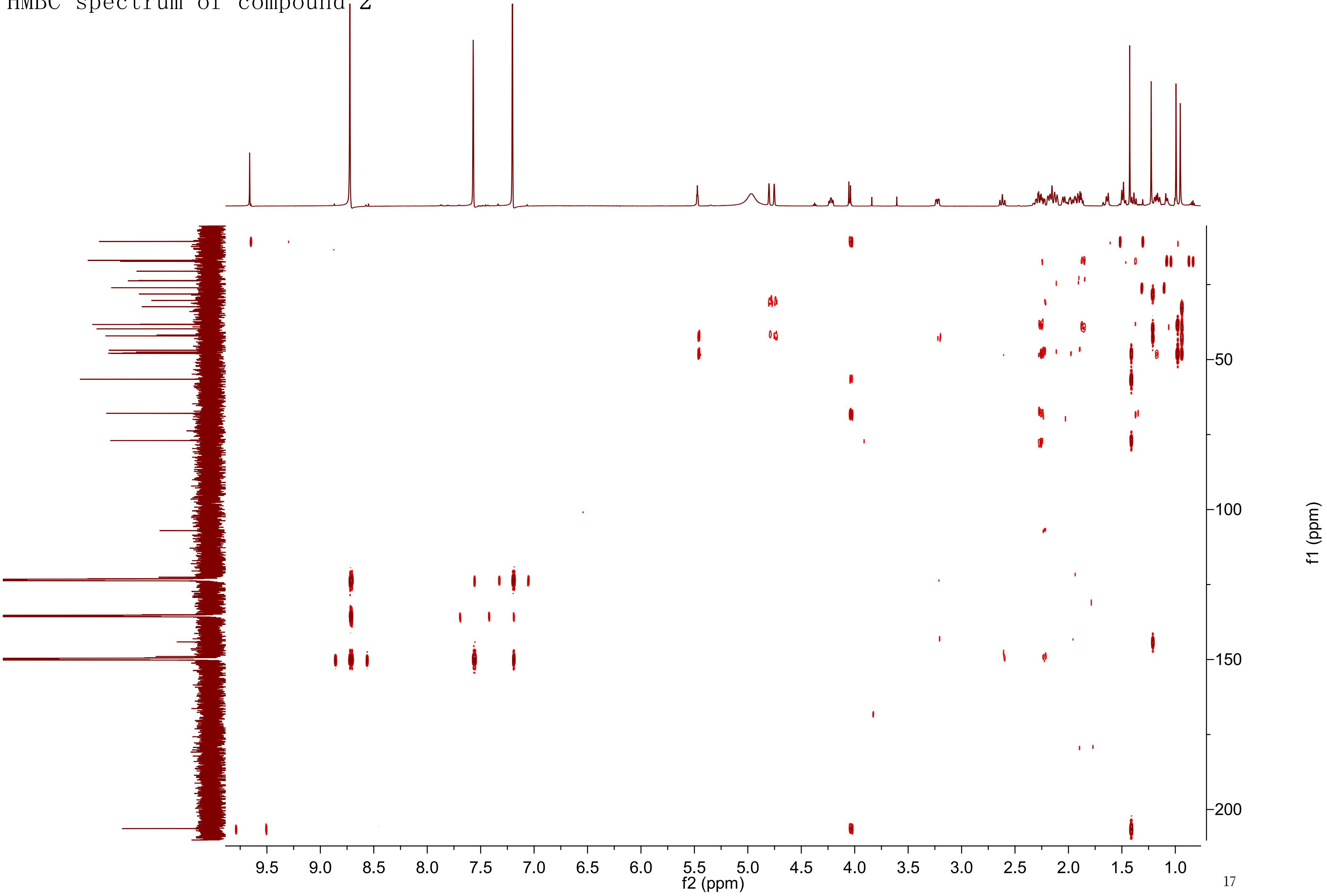

NOESY spectrum of compound **2**

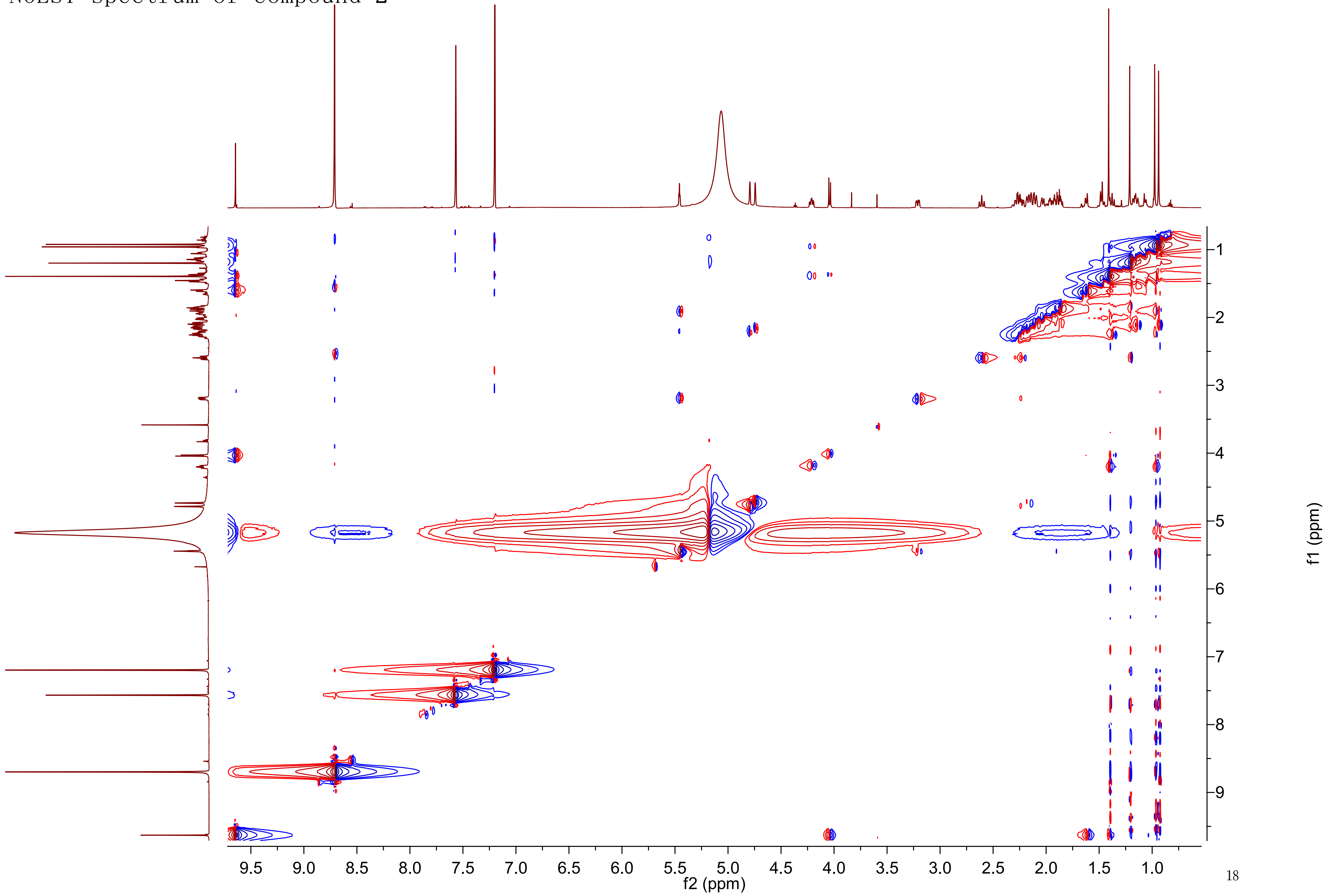

Supplement: Supplementary file 1 [file molecules-19-04301-s001.pdf]
